# Molecular profiling of metastatic breast cancer and target-based therapeutic matching in an Asian tertiary phase I oncology unit

**DOI:** 10.3389/fonc.2024.1342346

**Published:** 2024-05-15

**Authors:** Robert John Walsh, Rebecca Ong, Seng Wee Cheo, Peter Q.J. Low, Aishwarya Jayagopal, Matilda Lee, Natalie Ngoi, Samuel G. Ow, Andrea L.A. Wong, Siew Eng Lim, Yi Wan Lim, Valerie Heong, Raghav Sundar, Ross A. Soo, Cheng Ean Chee, Wei Peng Yong, Boon Cher Goh, Soo Chin Lee, David S.P. Tan, Joline S.J. Lim

**Affiliations:** ^1^ Department of Haematology-Oncology, National University Cancer Institute, Singapore, National University Health System, Singapore, Singapore; ^2^ Yong Loo Lin School of Medicine, National University of Singapore, Singapore, Singapore; ^3^ Department of Information Systems and Analytics, School of Computing, National University of Singapore, Singapore, Singapore; ^4^ Department of Medical Oncology, Tan Tock Seng Hospital, Singapore, Singapore; ^5^ Cancer and Stem Cell Biology Program, Duke-NUS Medical School, Singapore, Singapore; ^6^ The N.1 Institute for Health, National University of Singapore, Singapore, Singapore; ^7^ Singapore Gastric Cancer Consortium, Singapore, Singapore; ^8^ Cancer Science Institute, National University of Singapore, Singapore, Singapore; ^9^ National University of Singapore (NUS) Centre for Cancer Research (N2CR), Yong Loo Lin School of Medicine, National University of Singapore, Singapore, Singapore

**Keywords:** breast cancer, precision oncology, molecular profiling, next-generation sequencing (NGS), phase I

## Abstract

**Introduction:**

Molecular profiling of metastatic breast cancer (MBC) through the widespread use of next-generation sequencing (NGS) has highlighted actionable mutations and driven trials of targeted therapy matched to tumour molecular profiles, with improved outcomes reported using such an approach. Here, we review NGS results and treatment outcomes for a cohort of Asian MBC patients in the phase I unit of a tertiary centre.

**Methods:**

Patients with MBC referred to a phase I unit underwent NGS via Ion AmpliSeq Cancer Hotspot v2 (ACH v2, 2014–2017) prior to institutional change to FoundationOne CDx (FM1; 2017–2022). Patients were counselled on findings and enrolled on matched therapeutic trials, where available. Outcomes for all subsequent treatment events were recorded to data cut-off on January 31, 2022.

**Results:**

A total of 215 patients were enrolled with successful NGS in 158 patients. The PI3K/AKT/PTEN pathway was the most altered with one or more of the pathway member genes *PIK3/AKT/PTEN* affected in 62% (98/158) patients and 43% of tumours harbouring a *PIK3CA* alteration. Tumour mutational burden (TMB) was reported in 96/109 FM1 sequenced patients, with a mean TMB of 5.04 mt/Mb and 13% (12/96) with TMB ≥ 10 mt/Mb. Treatment outcomes were evaluable in 105/158 patients, with a pooled total of 216 treatment events recorded. Matched treatment was administered in 47/216 (22%) events and associated with prolonged median progression-free survival (PFS) of 21.0 weeks [95% confidence interval (CI) 11.7, 26.0 weeks] *versus* 12.1 weeks (95% CI 10.0, 15.4 weeks) in unmatched, with hazard ratio (HR) for progression or death of 0.63 (95% CI 0.41, 0.97; p = 0.034). In the subgroup of *PIK3/AKT/PTEN*-altered MBC, the HR for progression or death was 0.57 (95% CI 0.35, 0.92; p = 0.02), favouring matched treatment. Per-patient overall survival (OS) analysis (n = 105) showed improved survival for patients receiving matched treatment *versus* unmatched, with median OS (mOS) of 30.1 *versus* 11.8 months, HR = 0.45 (95% CI 0.24, 0.84; p = 0.013). Objective response rate (ORR) in the overall population was similar in matched and unmatched treatment events (23.7% *versus* 17.2%, odds ratio of response 1.14 95% CI 0.50, 2.62; p = 0.75).

**Conclusions:**

Broad-panel NGS in MBC is feasible, allowing therapeutic matching, which was associated with improvements in PFS and OS.

## Introduction

Precision oncology has drastically changed treatment paradigms in many solid organ tumours over the last decade, driven by an exponential increase in our knowledge of somatic molecular aberrations and the ability to target key oncogenic drivers. Knowledge of such aberrations is facilitated by the widespread adoption of next-generation sequencing (NGS) for somatic profiling, allowing molecularly targeted, or “matched”, therapies to be administered to patients more frequently. Precision oncology has seen great success in non-small cell lung carcinoma (NSCLC), with current National Comprehensive Cancer Network (NCCN) and European Society for Medical Oncology (ESMO) Precision Medicine Working Group guidelines recommending testing for actionable alterations via a broad-panel approach in newly diagnosed advanced NSCLC (1, 2).

Beyond NSCLC, organ-specific approvals for targeted therapy based on somatic sequencing results exist in a growing number of settings with targets including fibroblast growth factor receptor (FGFR), RAS/RAF, the homologous recombination repair (HRR) pathway, and isocitrate dehydrogenase 1 (IDH1) (3–7). Agnostic indications are increasing, with pembrolizumab approved in 2017 for use in pre-treated tumours exhibiting microsatellite instability (MSI; MSI-high) or deficiency in mismatch repair (dMMR) proteins and more recently in cases of high tumour mutational burden (TMB ≥ 10) (8, 9). Beyond immune checkpoint inhibition, in 2022, dabrafenib combined with trametinib was approved in advanced malignancies harbouring a *BRAF* V600E mutation, while multiple single-arm basket trials of the TRK inhibitors larotrectinib and entrectinib in *NTRK* fusion-positive solid tumours led to their approval in 2018 and 2019, respectively (10–12).

Metastatic breast cancer (MBC) has traditionally had limited utility for broad-panel somatic molecular profiling, with treatment decisions driven by hormone receptor status and the presence or absence of CerbB2 overexpression/amplification. Poly (ADP-ribose) polymerase (PARP) inhibitors olaparib and talazoparib improve progression-free survival (PFS) over standard-of-care chemotherapy in germline *BRCA1/2*-altered human epidermal growth factor receptor 2 (HER2)-negative MBC, which accounts for approximately 10% of MBC cases (13, 14). Both agents received subsequent approval; however, to date, this does not extend to their use in patients with somatic *BRCA1/2* mutations alone, though early data have suggested a potential role in this context (15).

Multiple trials have attempted to target mutations in *PIK3CA*, the gene encoding phosphatidylinositol 3-kinase (PI3K) catalytic subunit, which is altered in approximately 40% of MBC. Most notably in 2019, the phase III SOLAR 1 trial reported that alpelisib in combination with fulvestrant led to improved PFS over fulvestrant alone in hormone receptor positive, HER2-negative MBC harbouring hotspot mutations in *PIK3CA*, leading to the subsequent approval for this combination (16). Further studies have looked to target PI3K pathway alterations beyond *PIK3CA*. Both the phase II FAKTION and phase III CAPItello-291 trials have examined the addition of capivasertib, an oral inhibitor of AKT, to fulvestrant in patients with oestrogen receptor (ER)-positive, HER2-negative MBC after progression or relapse on an aromatase inhibitor (17, 18). The FAKTION authors reported a significant PFS and overall survival (OS) benefit favouring the novel combination; however, subgroup analysis showed that this benefit was primarily in patients considered to have PIK3/AKT/PTEN pathway alteration, with no significant benefit seen in non-altered tumours (17). Results of CAPItello-291 support a significant PFS benefit in the overall patient population with the addition of capivasertib to fulvestrant, with HR for progression or death of 0.60 [95% confidence interval (CI) 0.51, 0.71], although the magnitude of benefit was greater in those with AKT pathway alterations, HR = 0.50 (95% CI 0.38, 0.65) (18).

Phase I studies have provided evidence that molecularly matched therapy can lead to a similar benefit in refractory MBC, with several non-randomised studies showing the administration of matched therapy to be associated with improved outcomes (19–21). In a retrospective analysis including only MBC (n = 97), patients on matched clinical trials had improved PFS (HR = 0.52, p = 0.003) and OS (HR = 0.54, p < 0.001) *versus* those on non-matched trials (19).

Here, we report a retrospective analysis of MBC patients enrolled in the Integrated Molecular Analysis of Cancer (IMAC) study from April 28, 2014, to January 31, 2022. The IMAC study is an ongoing prospective trial using broad-panel sequencing of refractory solid-organ malignancies to identify targetable molecular alterations in the Phase I unit of the National University Cancer Institute, Singapore (NCIS). Earlier results detailing the feasibility and outcomes from April 28, 2014, to September 1, 2016, have been reported (22). We present broad-panel sequencing results as well as clinical outcomes of future treatment lines after administration of molecularly matched or unmatched therapy.

## Methods

### Patient selection

Patients with MBC enrolled in the IMAC program from April 28, 2014, to January 31, 2022, were included in the current retrospective analysis. The IMAC study enrolled patients with advanced solid organ malignancies reviewed in the Developmental Therapeutic Unit of NCIS. Available tumour tissue was obtained for NGS based on paraffin-embedded primary tumour and/or metastatic specimens, which were obtained during routine clinical care (e.g., surgery, biopsy or ascites, and pleural fluid drain). Wherever possible, fresh tumour samples were collected, with archival tumours utilised when a fresh sample was not available or technically feasible to obtain. Additionally, commencing from 2017, blood samples for circulating tumour (ct) DNA analysis were eligible for testing at the discretion of the patient’s primary physician should there be insufficient tumour tissue. Patients with successful sequencing were included in the mutational profile analysis of this current retrospective report.

### Sequencing and analysis

For patients enrolled from April 2014 to July 2017, sequencing was via amplicon-based Ion AmpliSeq Cancer Hotspot v2 (50 genes, **Appendix A**), performed in our institution on Ion Torrent/PGM System (Life Technologies, Camarillo, CA, USA) as previously described (22). Patients enrolled after August 2017 to January 31, 2022, underwent tumour sequencing via FoundationOne CDx (FM1) after an institutional change in panel use. Formalin-fixed, paraffin-embedded (FFPE) tissue was processed per institutional practice, and slides were submitted to Foundation Medicine (Cambridge, MA, USA) for testing. If adequate tissue was not available in patients with no plans for further biopsy, two tubes of whole blood were submitted to Foundation Medicine for analysis under the FoundationOne Liquid CDx platform. Variants identified by FoundationOne CDx are represented as pathogenic or likely pathogenic and variants of unknown significance. For the purposes of this analysis, the variants of uncertain significance were excluded. FoundationOne CDx specimens were also simultaneously profiled for TMB as well as MSI status (23).

### Treatment selection

Patient sequencing results were discussed in the NCIS Molecular Tumour Board to identify potential matched therapies or clinical trials. Patients were counselled on outcomes of sequencing and molecular tumour board discussion and enrolled on matched trials, if available. All patients provided informed consent prior to joining a given clinical trial, all of which were approved by the appropriate local Research and Ethics Committee. If matched trials were not available in the unit, patients could undergo matched treatment off trial or unmatched treatment on or off trial after discussion with their treating oncologist.

### Definition of matched treatment

Treatment was defined as matched if a molecular alteration detected on sequencing was targeted, or within the pathway targeted, by the administered drug. Treatment was considered non-matched if no molecular alterations were detected or if alterations detected, or the pathways within which they lie, were not targeted by administered treatment. The expression status of ER/progesterone receptor (PR) and CerbB2 was not taken into consideration. Specific scenarios included the following:

- Anti-HER2 therapy is considered matched if *ERBB2* alteration or amplification was detected on sequencing analysis regardless of immunohistochemistry results.
- Therapeutics targeting the DNA damage response (DDR) pathway including PARP inhibitors and ATR inhibitors would be considered matched in the presence of mutations in the following list of homologous recombination-related genes previously described by Tung et al. (15): *ATM*, *ATR*, *BAP1*, *BARD1*, *BLM*, *BRIP1*, *CHEK1*, *CHEK2*, *CDK12*, *FANCA*, *FANCC*, *FANCD2*, *FANCF*, *MRE11A*, *NBN*, *PALB2*, *RAD50*, *RAD51C*, *RAD51D*, or *WRN*. Platinum therapy was not considered matched in the presence of such mutations.
- Immunotherapy was considered matched in the presence of high TMB (≥10) or MSI-high/dMMR.
- Patients with detected alterations (including non-hotspot *PIK3CA* mutations) in any of *PI3K*, *AKT*, and/or *PTEN* were considered as *PI3K/AKT/PTEN* altered, and treatment targeting this pathway was recorded as matched.

### Follow-up

Patients enrolled on clinical trials underwent follow-up and tumour response assessment per trial protocol, while those treated outside of clinical studies were followed up per standard institution clinical practice.

### Evaluable patients

Patients with successful NGS profiling were included in baseline characteristic assessment and mutational profile analysis. Patients who commenced on a new line of systemic therapy after sequencing results were available, with completion of at least one treatment cycle by data cut-off (January 31, 2022), were considered evaluable for clinical outcomes. Patients with less than one completed treatment cycle but with objective evidence of a disease progression event were deemed evaluable. Those lost to follow-up or continuing on treatment without disease progression at data cut-off were censored at the last documented clinic review.

### Statistical analysis

Baseline patient characteristics were tabulated and summarised with descriptive statistics. PFS was calculated from the date of first administration of the drug to the date of radiological or clinical progression or death from any cause. Response assessment was per Response Evaluation Criteria in Solid Tumors (RECIST) version 1.1 criteria. For patients with multiple sequential treatment events after sequencing, details of each individual treatment event (including matched status) were recorded and used to calculate a pooled progression-free survival analysis. The hazard ratio (HR) for risk of progression or death was calculated for this pooled assessment with appropriate frailty adjustment for shared identity.

A per-patient analysis of OS (calculated from the date of treatment start post-sequencing to death or censoring) by matched status was performed. Overall survival of patients receiving matched therapy at any point post-sequencing was compared to that of patients never receiving matched treatment post-sequencing. Clinical benefit rate was calculated as the proportion of patients with complete or partial response or more than 24 weeks of stable disease. Statistical analysis and KM plot generation was performed using STATA version 17.0 (STATA Corp., College Station, TX, USA). Oncoplot and Swimmer plot generated using R and R studio (URL https://www.R-project.org/, Boston, MA; URL http://www.rstudio.com/).

## Results

A total of 215 patients with MBC were enrolled on IMAC from April 28, 2014, to January 31, 2022. Sequencing was successful in 73% of patients (158/215), with 105 patients evaluable for treatment response (66%, 105/158). Baseline characteristics of sequenced patients (n = 158) are summarised in **Table 1**.

**Table 1:** Baseline characteristics of sequenced patients (n = 158).

Characteristic	Number (%)
Mean age (range)	55 (29–76)
Female	158 (100)
*De novo* metastatic	69 (43.7)
Ethnicity	
Chinese	102 (64.6)
Malay	24 (15.2)
Indian	5 (3.16)
Other	27 (17.1)
Sequencing platform	
FoundationOne*	109 (69.0)
AmpliSeq Cancer Hotspot v2	49 (31.0)
Tissue sample	
Archival	86 (54.4)
Fresh	67 (42.4)
Blood	5 (3.2)
Receptor status	
ER/PR positive, HER2 negative	95 (60.1)
HER2 positive	28 (17.7)
TNBC	35 (22.2)
Prior therapy for MBC	
Median number of prior lines (range)	3 (0–13)
Chemotherapy	129 (81.6)
Endocrine therapy	97 (61.4)
CDK 4/6 inhibitor	54 (34.2)
Immune checkpoint inhibitor	15 (9.49)
PI3K/mTOR/AKT inhibitor	16 (10.1)
PARP inhibitor	2 (1.27)

CDK, cyclin-dependent kinase; ER, oestrogen receptor; MBC, metastatic breast cancer; PR, progesterone receptor; HER2, human epidermal growth factor receptor 2; PARP, poly-ADP ribose polymerase; PIK3, phosphoinositide 3-kinase; TNBC, triple-negative breast cancer.

*Includes both formalin-fixed, paraffin-embedded (FFPE) tissue and liquid analysis.

### Tumour profiling outcomes

**Figure 1:**
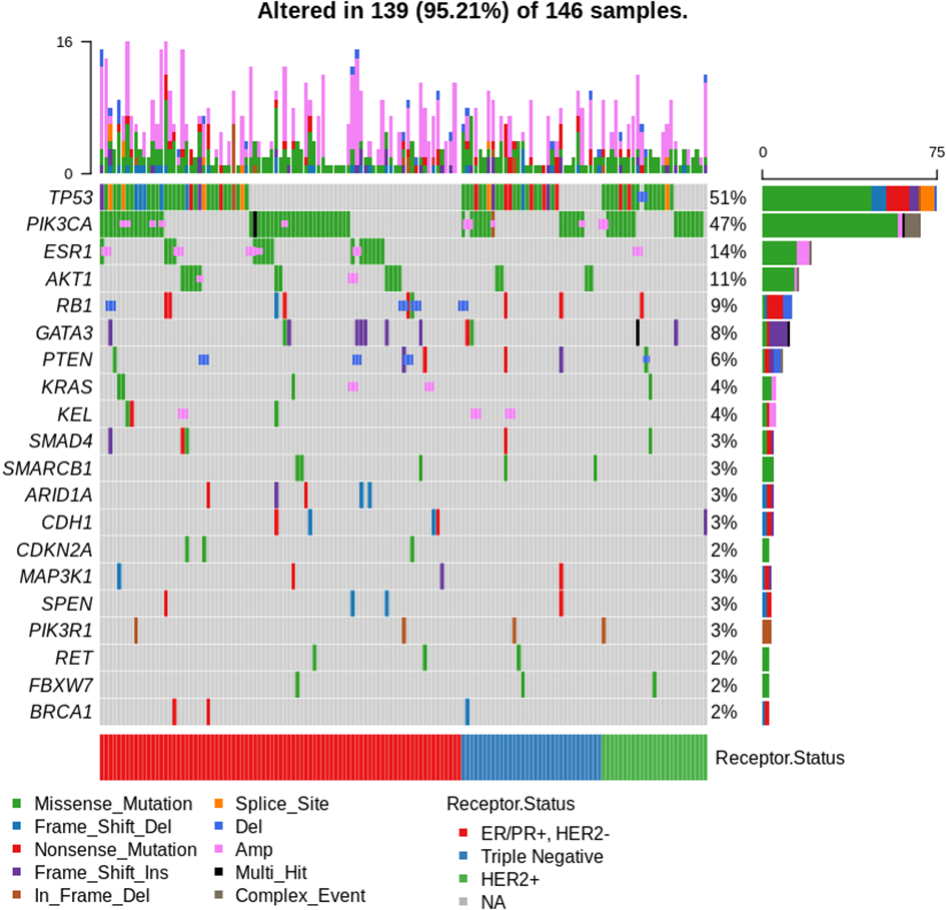
Oncoplot of results for patients with successful sequencing (n = 158). Top 20 most frequently mutated genes shown. A total of 12 patients had no mutations identified. Arranged by oestrogen receptor (ER), progesterone receptor (PR), and human epidermal growth factor receptor 2 (HER2) status.

**Figure 1** shows an oncoplot of molecular profile results of patients with successful sequencing (n = 158). *TP53* was the most commonly mutated gene (47%, 75/158 patients), with a significantly higher frequency of *TP53* alterations seen in ER-negative *vs.* ER-positive tumours (65 *vs.* 38%, p = 0.001). *PIK3CA* alterations were seen in 43% (68/158) of patients, with 17 patients having more than one *PIK3CA* mutation. Of these, the hotspot mutation at *PIK3CA* H1047R was most common, identified in 17% (27/158) of tumours.

The most commonly altered pathway was PI3K/AKT/PTEN, with alterations in one or more of the pathway member genes (*PIK3/AKT/PTEN*) seen in 62% (98/158) of patients, with no difference in frequency in ER-positive *versus* ER-negative tumours (p = 0.86) or sequencing performed on archival *versus* fresh samples (p = 0.229). Aberrations in the DDR pathway were present in 10% (16/158) of sequenced patients, with 5/158 patients harbouring *BRCA1* mutations and 3/158 *BRCA2* alterations. One patient had co-occurring *BRCA1* and *BRCA2* mutations (*BRCA1* S573*, *BRCA2* A938fs*21).

Of 109 patients sequenced on the FoundationOne CDx platform, TMB was reported in 96 cases, with a mean TMB of 5.04 mt/Mb (range 0.0–40 mt/Mb). High TMB (≥10 mt/Mb) was seen in 12.5% of patients (12/96). No MSI-high cases were detected.

Germline testing was performed in 26% (41/158) of sequenced patients, with pathogenic or likely pathogenic alterations identified in 8/41 cases. Patients with germline alterations who underwent sequencing with FM1 (5/8) had a corresponding somatic mutation identified in all cases. Three patients with known germline mutations in either *BRCA1/2* had tumour profiling performed on the AmpliSeq Cancer Hotspot V2 test panel, which did not include *BRCA1/2*, and thus, somatic *BRCA* alterations were not identified.

### Treatment event outcomes

**Figure 2:**
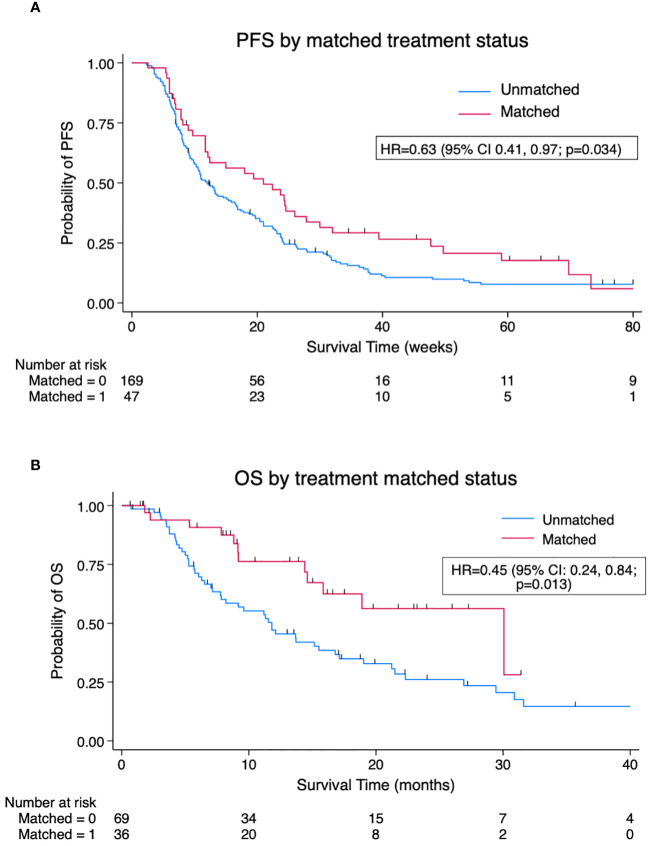
**(A)**. Kaplan–Meier pooled analysis of progression-free survival (PFS) by matched *versus* unmatched treatment (n = 216 treatment events). **(B)** Kaplan–Meier per-patient analysis of overall survival (OS) by matched *versus* unmatched status of post-sequencing treatment (n = 105 patients). Survival favours patients receiving matched therapy at any point post-sequencing *versus* those never receiving matched therapy. HR, hazard ratio.

A total of 105 patients (**Supplementary Table 3**) underwent evaluable treatment after sequencing and had a total of 216 treatment events during the follow-up period, of whom 47/216 (22%) were genomically matched. Matched events had a significantly improved median PFS of 21.0 weeks (95% CI 11.7, 26.0 weeks) *versus* 12.1 weeks (95% CI 10.0, 15.4 weeks) in unmatched with HR for progression of 0.63 (95% CI 0.41, 0.97; p = 0.034) (**Figure 2A**). Adjusting for the treatment line gave an HR = 0.59 (95% CI 0.37, 0.96; p = 0.035), favouring matched treatment. A per-patient analysis (n = 105) of OS based on the matched status of treatment post-sequencing was performed. A significant benefit was seen for patients receiving matched treatment at any point post-sequencing *versus* those not receiving matched treatment, with median OS (mOS) 30.1 *versus* 11.8 months, HR = 0.45 (95% CI 0.24, 0.84; p = 0.013) (**Figure 2B**). Clinical benefit rate was superior with matched therapy, 46.8% (22/47) *versus* 29.6% (50/169), odds ratio (OR) 2.09 (95% CI 1.08, 4.05; p = 0.028), but no significant difference was seen in terms of objective response rate (ORR) of 23.7% for matched treatment *versus* 17.2% for unmatched treatment, OR 1.14 (95% CI 0.50, 2.62; p = 0.752).

### Treatment events in *PIK3/AKT/PTEN*-altered patients

Alterations in *PIK3/AKT/PTEN* were seen in 64% (67/105) of sequenced patients who underwent evaluable treatment. The majority of these treated *PIK3/AKT/PTEN*-altered patients were HER2 negative (84%, 56/67). The 67 treated *PIK3/AKT/PTEN*-altered patients had 137 evaluable treatment events, of which 42/137 (31%) were genomically matched. The majority of these 42 matched events, 25/42, involved the administration of treatment targeting the *PIK3/AKT/PTEN* pathway (see **Supplementary Table 1**), while 12/42 were matched to anti-HER2 therapy in the presence of a coexisting *ERBB2* amplification.

**Figure 3:**
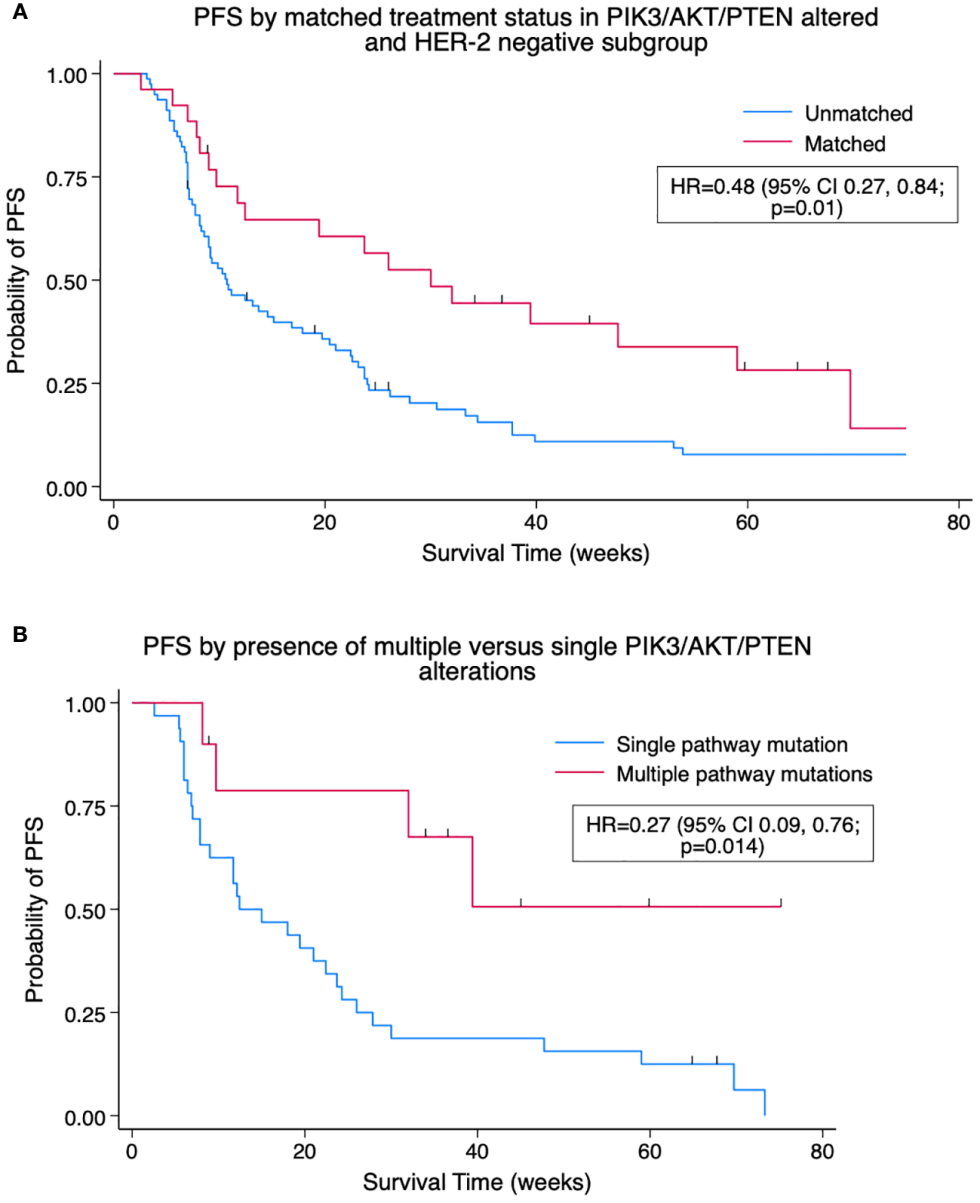
**(A)** Kaplan–Meier pooled analysis of progression-free survival (PFS) by matched treatment status in *PIK3/AKT/PTEN*-altered and HER2-negative subgroup. HR, hazard ratio; HER2, human epidermal growth factor receptor 2. **(B)** Kaplan–Meier pooled analysis of PFS by presence of multiple *versus* single *PIK3/AKT/PTEN* alterations for patients with at least one *PIK3/AKT/PTEN* alteration on matched therapy. HR, hazard ratio.

Matched treatment events in *PIK3/AKT/PTEN*-altered patients were associated with improved median PFS compared to unmatched treatment events, 19.4 *versus* 10.6 weeks, HR = 0.57 (95% CI 0.35, 0.92; p = 0.02). The difference was more pronounced in a subgroup of *PIK3/AKT/PTEN* altered, HER2-negative/*ERBB2* non-altered patients, where matched treatment events displayed prolonged median PFS of 30.0 *versus* 10.7 weeks, HR = 0.48 (95% CI 0.27, 0.84; p = 0.01). Of the 42 *PIK3/AKT/PTEN*-altered patients matched to therapy, 10 (24%) had more than one *PIK3/AKT/PTEN* alteration. Patients with multiple *PIK3/AKT/PTEN* alterations had an improved PFS on matched therapy (median PFS not reached) *versus* those with single aberrations (12.4 weeks), HR = 0.27 (95% CI 0.09, 0.76; p = 0.014). The Kaplan–Meier plots are shown in **Figure 3**.

### DDR pathway alterations

Of patients with DDR pathway mutations identified, 12/16 underwent evaluable treatment post-sequencing with a pooled total of 26 treatment events. There was no significant difference in PFS in patients with DDR pathway mutations (median PFS = 16.4 weeks) *versus* those without (median PFS = 12.4 weeks), HR = 0.71 (95% CI 0.38, 1.32, p = 0.282). Matched treatment was recorded for three patients with DDR pathway mutations. Olaparib was administered in combination with intrathecal methotrexate in a patient with germline (and corresponding somatic) *BRCA1* pathogenic variant with a PFS of 59 weeks and as a single agent in a patient with germline (and somatic) *PALB2* alteration for 24 weeks prior to progression. Treatment with an ATR inhibitor in the setting of a clinical trial gave a relatively short PFS of 12 weeks in a patient with somatic *BRCA1* Q544* (no germline testing performed). One patient received PARPi prior to study enrolment.

### TMB and immune checkpoint inhibitors

**Figure 4:**
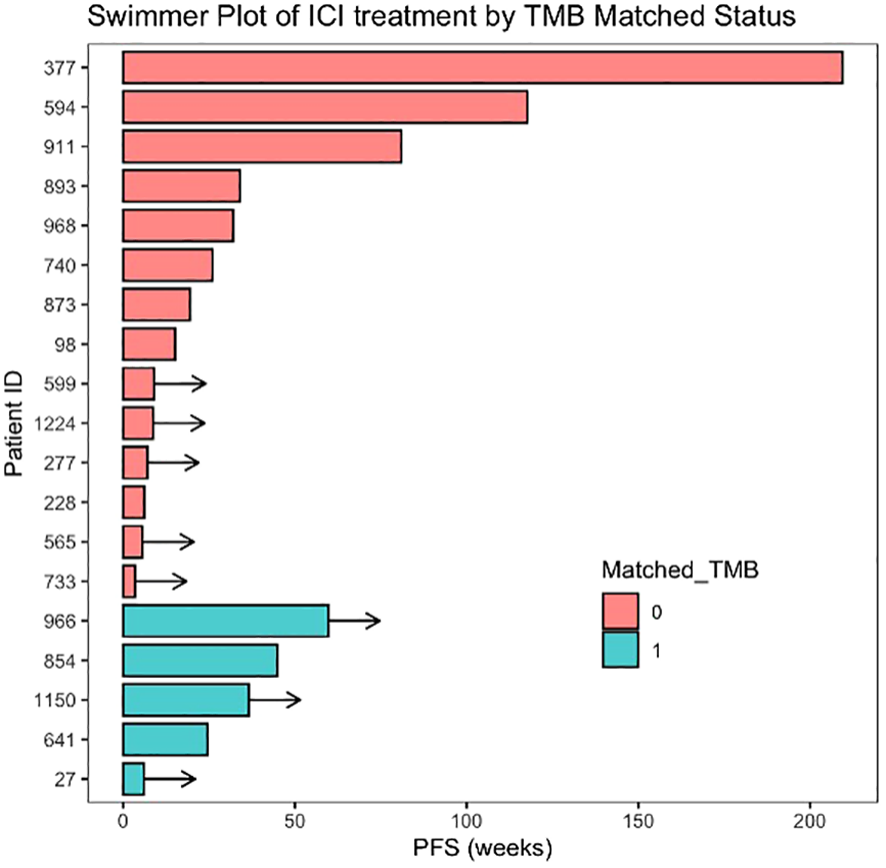
Swimmer plot of patients treated with immune checkpoint inhibitors (ICIs) arranged by matched status. Matched TMB = 1 represents patients matched to ICI therapy based on the presence of high tumour mutational burden (TMB ≥ 10 mt/Mb). Arrows indicate ongoing treatment at time of data cut-off.

Immune checkpoint inhibitors (ICIs) were administered alone or in combination to 19 patients (**Supplementary Table 2**), eight of whom received the combination of anti-PD-L1, AKT inhibitor, and a PARP inhibitor as part of an ongoing clinical trial. These eight patients harboured a mutation in one of *PTEN*/*AKT* and/or *PIK3CA* and were therefore considered matched to the use of an AKT inhibitor. In 5/19 ICI-treated patients, TMB was ≥10, and treatment was considered TMB matched. Median PFS in the 19 ICI-treated patients was 26.0 weeks (95% CI 7, -) with eight patients continuing treatment at the point of data collection (**Figure 4**). Analysis of this ICI-treated cohort by TMB-matched status showed no significant difference with a median PFS (mPFS) of 44.9 *vs.* 32.0 weeks in TMB-matched *vs.* unmatched patients, HR = 0.59 (95% CI 0.12, 2.94; p = 0.52).

## Discussion

In this retrospective study of a cohort of MBC patients from a tertiary oncology centre in Asia, we report a higher PFS for patients receiving matched *versus* unmatched treatment, a finding consistent with prior publications (19, 24, 25). Median PFS in the current study was 21.0 weeks in pooled treatment event analysis, a value that is difficult to compare across publications of early-phase trial cohorts due to the heterogeneous nature of such populations. A prior publication on the IMAC study in 2018, including multiple tumour types, reported a median PFS of 2.9 months, similar to the 3.2 months reported by O’Carrigan and colleagues in a pre-treated MBC cohort (19, 22). Our study also yielded a significant improvement in OS in patients receiving matched therapy at any point post-sequencing *versus* those who do not go on to receive any matched treatment post-sequencing, with HR = 0.45 (95% CI 0.24, 0.84; p = 0.013), highlighting that matching patients based on sequencing outcomes remain beneficial even if the matching cannot occur in the immediate setting. It is therefore vital for specialised drug development units to keep track of patients with targetable molecular mutations and update primary oncologists on new treatment options for matching when available. While OS analysis may be affected by subsequent treatment received and the presence of censored individuals in both matched and unmatched groups, it remains an encouraging result reinforcing the potential benefit of matched therapy in MBC. The frequency of clinical benefit rate (CBR) in the current study was higher for matched treatment events (OR 2.09, p = 0.028), although response rates were similar regardless of matched status at 23% (matched) *versus* 21% (unmatched) and were overall in keeping with expected responses for pre-treated MBC.

Of all pooled treatment events, 22% were matched based on sequencing results, with the most frequent target being the *PIK3/AKT/PTEN* alterations, which were present in 64% of patients. This matching frequency is lower than reported in the pan-cancer IMPACT trial (54%) and in an MBC cohort by O’Carrigan et al. (83%) but similar to that of the Moores Cancer Center PREDICT analysis (20, 26). The frequency of matching increased over time, with 11% of treatment events in the first 70 enrolled patients being matched, increasing to 22% for the second 70 patients enrolled and 35% for the most recently recruited group. This may be attributed to the developing nature of the NCIS Developmental Therapeutic Unit over the trial period and a corresponding increase in the number of potential trial options available along with increasing access to approved biomarker-selected treatments such as alpelisib in *PIK3CA* hotspot mutated tumours. The reported rate in the current study is also contributed by the pooling of all treatment events post-sequencing. If only the first line post-sequencing were examined, the number of patients matched to therapy was 30.5% (32/105) *versus* 22% if all treatment events were considered.

*PIK3*, *AKT*, and *PTEN* were frequently mutated in the current cohort. Currently approved therapy targeting this pathway includes alpelisib in combination with fulvestrant; however, its use is restricted to patients with HER2-negative MBC harbouring one of 11 *PIK3CA* hotspot mutations, based on inclusion criteria of the phase III SOLAR-1 trial (16). However, the phase II FAKTION trial points to the benefit of pathway-targeted therapy for patients with a wider span of mutations affecting *PIK3/AKT/PTEN* when treated with capivasertib in combination with fulvestrant (17). More recently, phase III CAPItello-291 has shown PFS benefit for unselected MBC after progression on aromatase inhibitor with the addition of capivasertib to fulvestrant with the magnitude of benefit more pronounced in AKT pathway-altered patients (18). Results from our current study support the use of matched therapy in a wider pool of *PIK3/AKT/PTEN*-altered cases with PFS improved in such patients receiving matched treatment compared to those on unmatched therapy (HR = 0.57, p = 0.02). Of patients receiving therapy targeting the PI3K/AKT/PTEN pathway, nine had mutations outside *PIK3CA* hotspot alterations, with four of nine (44%) having a PFS of ≥12 weeks on matched therapy. Of those matched to therapy, the benefit was enhanced in patients with multiple alterations in *PIK3*, *AKT*, or *PTEN versus* those with single mutations, with HR for progression or death of 0.27 (**Figure 3B**). This supports previous reports of increased ORR and PFS in patients with multiple PIK3Ca mutations while ongoing treatment with fulvestrant and taselisib (27). This highlights the potential for patients with non-hotspot mutations to benefit from treatment targeting the PI3K/AKT/PTEN pathway and the role that broad-panel NGS has in identifying such alterations.

The number of patients with mutations in the DDR pathway was low (16/158), restricting any analysis of outcomes by treatment status. One patient (IMAC 660, **Supplementary Table 1**) with a somatic and germline-detected *PALB2* alteration had a PFS of 24 weeks with olaparib despite being heavily pre-treated (eight lines prior to therapy). Although anecdotal, this case highlights the expanded role of PARP inhibition outside of germline *BRCA1/2*-altered HER2-negative MBC. The benefit of PARPi outside of current approval limits has been reported in a phase I trial combining olaparib and capivasertib as well as phase II single-agent trials TBCRC-048 and Talazoparib Beyond BRCA (15, 28, 29).

We report a subgroup of patients who received ICI treatment for MBC (19/105 patients) of whom five had high TMB. The mPFS of 26 weeks in this ICI-treated group is promising when compared to those of trials of enriched subgroups, such as KEYNOTE-158, which reported an mPFS of 4.1 months in non-colorectal MSI-high cancers treated with pembrolizumab, and suggests that there remains a small group of MBC patients who can gain significant benefit from ICI (9). The role of immunotherapy in MBC is currently limited to first-line indication for pembrolizumab in combination with chemotherapy in PD-L1-positive, treatment-naïve triple-negative breast cancer (TNBC) in combination with chemotherapy, whereas its use in later-line settings as single-agent therapy has not shown significant improvement in treatment outcomes (30, 31). In the treatment-refractory setting, pembrolizumab has agnostic approval in the presence of dMMR/MSI-H or TMB ≥ 10 mt/Mb (8, 9). Numerous ongoing studies continue to evaluate the application of ICI in MBC while attempting to derive an optimal biomarker (32).

Our study has several strengths and limitations. This is one of the first reports of sequencing results and clinical outcomes in an Asian-predominant population and was set within a tertiary referral centre, which enabled access to a range of novel targeted therapies. This is of importance given the number of prospective clinical trials that are being carried out, at least in part, in Asian cancer centres and allows investigators to contextualise the molecular profile of breast cancer in this region. Sequencing was performed on validated commercial platforms ensuring robust reproducible results obtained.

The pooled analysis of treatment events risks incorporating bias where patients receive multiple subsequent treatment lines, each analysed as an individual event. This was adjusted using the frailty adjustment for shared identity in STATA analysis. The number of patients enrolled thus far has not allowed for powering to explore matching at each treatment line, which may be of interest in future studies when larger cohorts of patients with longer-term follow-up are available for analysis. The retrospective nature of the analysis risks selection bias, and low numbers of DDR pathway mutations are in part a reflection of the sample size and restrict any comparative assessment of this subgroup. The use of a more restricted sequencing panel prior to the institutional change to FM1 means that specific alterations will have been unidentified and underreported, including somatic *BRCA1/2* mutations, as these genes were not included in the 50 gene panel list of ACH v2 (**Supplementary Appendix A**). Similarly, TMB and MSI status were not assessed by ACH v2. Mutation detection may have also been affected by tumour heterogeneity, which has been well-described in breast cancer (33, 34). The majority of patients in the current studied cohort underwent somatic sequencing on archival tissue. It is possible the mutational profile of a tumour may be significantly different at the point of study entry in comparison to the date of archival tissue acquisition due to the temporal evolution of genomic alterations. Furthermore, the spatial evolution of clones and sub-clones means that the biopsy site may have affected alterations detected in our cohort.

In conclusion, this study highlights the feasibility of somatic NGS in MBC in facilitating therapeutic matching, primarily in patients with *PIK3/AKT/PTEN* alterations, which were seen in the majority. Matched therapy is shown to be associated with an improvement in PFS and OS over unmatched treatment in this cohort.

## Data availability statement

The original contributions presented in the study are included in the article/**Supplementary Material**, further inquiries can be directed to the corresponding author/s.

## Ethics statement

The studies involving humans were approved by National Healthcare Group Domain Specific Review Board (NHG DSRB). The studies were conducted in accordance with the local legislation and institutional requirements. The participants provided their written informed consent to participate in this study.

## Author contributions

RW: Conceptualization, Data curation, Formal analysis, Investigation, Methodology, Project administration, Visualization, Writing – original draft, Writing – review & editing, Resources, Software. RO: Conceptualization, Data curation, Investigation, Methodology, Project administration, Writing – original draft, Writing – review & editing. SC: Data curation, Investigation, Methodology, Writing – review & editing, Conceptualization. PL: Data curation, Investigation, Methodology, Project administration, Resources, Writing – review & editing, Conceptualization. AJ: Formal analysis, Methodology, Project administration, Resources, Software, Writing – review & editing, Conceptualization. ML: Investigation, Methodology, Project administration, Resources, Writing – review & editing, Conceptualization. NN: Data curation, Investigation, Methodology, Project administration, Resources, Writing – review & editing, Conceptualization. SO: Investigation, Methodology, Project administration, Resources, Writing – review & editing, Conceptualization. AW: Investigation, Methodology, Project administration, Resources, Writing – review & editing, Conceptualization. SEL: Investigation, Methodology, Project administration, Resources, Writing – review & editing, Conceptualization. YL: Investigation, Methodology, Project administration, Resources, Writing – review & editing, Conceptualization. VH: Data curation, Investigation, Methodology, Resources, Writing – review & editing, Conceptualization. RS: Investigation, Methodology, Resources, Writing – review & editing, Conceptualization. RS: Investigation, Methodology, Project administration, Resources, Writing – review & editing, Conceptualization. CC: Investigation, Methodology, Project administration, Resources, Writing – review & editing, Conceptualization. WY: Investigation, Methodology, Project administration, Resources, Writing – review & editing, Conceptualization. BG: Conceptualization, Investigation, Methodology, Project administration, Resources, Supervision, Writing – review & editing. SL: Conceptualization, Investigation, Methodology, Project administration, Resources, Supervision, Writing – review & editing. DT: Conceptualization, Formal analysis, Funding acquisition, Investigation, Methodology, Project administration, Resources, Supervision, Validation, Visualization, Writing – original draft, Writing – review & editing. JL: Conceptualization, Data curation, Formal analysis, Funding acquisition, Investigation, Methodology, Project administration, Resources, Software, Supervision, Validation, Visualization, Writing – original draft, Writing – review & editing.
