# Supplementary material for: Molecular profiling of metastatic breast cancer and target-based therapeutic matching in an Asian tertiary phase I oncology unit
Source: Front Oncol. 2024 May 15;14:1342346. doi: 10.3389/fonc.2024.1342346 (PMC11133600; doi:10.3389/fonc.2024.1342346)
Supplement: Supplementary file 1 [file DataSheet_1.docx]

| **Study ID** | **Age** | **De-novo MBC** | **ER%** | **HER2** | **No. tx lines prior to enrolment** | **Sequencing platform** | **Sequencing results** | **TMB (mt/Mb)** | **Matched therapy^#^** | **Best response** | **PFS (weeks)** |
| --- | --- | --- | --- | --- | --- | --- | --- | --- | --- | --- | --- |
| 011 | 47 | Y | 30 | + | 9 | ACH v2 | PIK3CA E545K | NR | PIK3 alpha/beta inhibitor | PD | 6.86 |
| 043 | 67 | N | 10 | - | 4 | ACH v2 | PIK3CA A400V, H1047R  RET L923F NRAS_L19DfsTer12 | NR | PIK3 alpha/beta inhibitor | PR | 9.71 |
| 052 | 50 | N | 85 | - | 3 | FM1 | PTEN loss CDKN2A H83Y subclonal TP53 N288fs*18 MAP2K loss FGF19, FGF4 and FGF4 amplification (equivocal) EMSY amplification CCND1 amplification (equivocal) | NR | Fulvestrant + CDK 4/6 inhibitor + PIK3 alpha inhibitor | SD | 23.71 |
| 088 | 64 | N | 0 | - | 1 | ACH v2 | ATM F858L  PIK3CA I391M  TP53 L299RfsTer46 | NR | PIK3 alpha/beta inhibitor | PD | 8.14 |
| 121 | 44 | N | 80 | - | 6 | ACH v2 | PIK3CA_H1047R | NR | PIK3 alpha/beta inhibitor | PD | 7.86 |
| 149 | 61 | N | 10 | - | 3 | ACH v2 | PIK3CA E542K SMARCB1 T72K | NR | FGFR inhibitor | SD | 69.71 |
| 242 | 46 | N | 90 | - | 9 | FM1 | PTEN splice site 80-23_del  ESR1 D538G | NR | AKT inhibitor + fulvestrant | SD | 30.0 |
| 251 | 54 | Y | 100 | - | 4 | ACH v2 | PIK3CA H1047R | NR | PIK3 alpha/beta inhibitor | SD | 12.43 |
| 476 | 65 | N | 0 | + | 2 | FM1 | ERBB2 amplification – equivocal⧺ PIK3CA H1047R CDKN2B rearrangement exon 2 NOTCH2 deletion exons 3-27 TAF1 A19fs*50 TP53 E286* | 10 | Eribulin + trastuzumab | SD | 12.14 |
|  |  |  |  |  |  |  |  |  | STAT3 inhibitor+ trastuzumab | PD | 5.43 |
|  |  |  |  |  |  |  |  |  | Gemcitabine + carboplatin + trastuzumab | PD | 7.86 |
| 505 | 48 | N | - | - | 7 | FM1 | PALB2 splice site 2748+1G>A PIK3CA E110del, R88Q ESR1 D538G EPHB1 M447I | 16 | Fulvestrant + alpelisib | SD | **136*** |
| 511 | 36 | Y | 0 | - | 0 | FM1 | PIK3CA amplification – equivocal⧺ BRCA1 A322fs*19 CDC73 deletion exons 11-12 GATA3 E228* PRKCI amplification – equivocal⧺ RB1 splice site 1498+1G>C TERC amplification – equivocal⧺ TP53 S127F | 4 | Olaparib + intra-thecal methotrexate | SD | 59 |
| 565 | 63 | Y | 90 | - | 6 | FM1 | CCND1 Amp VEGFA amp  KDM5A amp WHSC1L1 amp PTEN loss FGF3 amp MDM2 amp ESR1 amp CDK4 amp BRAF amp FGFR1 amp FGF4 amp FGF 19 amp GATA3 p409fs*99 | NR | Anti-PD-L1 + AKT inhibitor + PARPi | PD | 5.57 |
| 599 | 54 | Y | 90 | - | 9 | FM1 | PTEN loss FGFR1 amplification – equivocal⧺ MDM2 amplification ABL1 K609del BCL2L1 amplification – equivocal⧺ FRS2 amplification RB1 S391* ZNF217 amplification | 9 | Anti-PD-L1 + AKT inhibitor + PARPi | PD | 9.0 |
| 660 | 42 | N | 80 | - | 8 | FM1 | AKT1 amplification - equivocal† CDK4 amplification ERBB2 amplification - equivocal† ESR1 Y537N PALB2 loss exons 12-13 KRAS amplification AURKA amplification ARFRP1 amplification BCL2L2 amplification GNAS amplification SOX9 E75K SPEN S1492fs*3 ZNF217 amplification | 9 | Olaparib | SD | 24.29 |
| 740 | 61 | N | 0 | - | 3 | FM1 | MYC amplification FGF12 amplification BCL2L1 amplification NOTCH3 amplification RAD21 amplification LYN amplification CBFB E84fs*32 PIK3CA E545K CASP8 P363fs*37 TP53 splice site 673-1G>C | 1.26 | Anti-PD-L1 + AKT inhibitor + PARPi | SD | 26 |
| 763 | 48 | N | 90 | - | 2 | FM1 | PIK3CA E542K GATA3 S438fs*39 | 1.26 | Fulvestrant + alpelisib | PD | 7.0 |
|  |  |  |  |  |  |  |  |  | Exemestane + everolimus | SD | 67.57 |
| 827 | 67 | Y | 90 | + | 8 | FM1 | PIK3C2B amplification MDM4 amplification ERBB2 amplification ESR1 amplification CCND1 amplification ZNF217 amplification FGF4 amplification FGF3 amplification FGF19 amplification GATA3 W329fs*23 TP53 R273P GATA3 P409fs*99 CBFB splice site 165+1G>C | 18.91 | Trastuzumab emtansine | SD | 22.43 |
|  |  |  |  |  |  |  |  |  | STAT3 inhibitor+ trastuzumab | PD | 6.0 |
|  |  |  |  |  |  |  |  |  | Pembrolizumab | PD | 6.0 |
|  |  |  |  |  |  |  |  |  | Capecitabine + lapatinib + trastuzumab | SD | 27.86 |
|  |  |  |  |  |  |  |  |  | Docetaxel + trastuzumab + pertuzumab | SD | 15.0 |
| 836 | 65 | N | 80 | + | 4 | FM1 | ERBB2 amplification GATA3 L355fs*17 MAP2K4 R287H PIK3CA H1047L | 3.78 | Capecitabine + trastuzumab | SD | 73.29 |
|  |  |  |  |  |  |  |  |  | Trastuzumab emtansine | SD | 11.71 |
|  |  |  |  |  |  |  |  |  | Tamoxifen + everolimus | SD | 18.0 |
| 841 | 69 | Y | 90 | - | 3 | FM1 | AKT1 E17K BCL2L1 amplification CDK4 amplification CDKN2A D108N SMAD4 D351H TP53 R333fs*12 | 7.57 | Exemestane + everolimus | SD | 11.71 |
| 856 | 54 | Y | 90 | - | 8 | FM1 | ESR1 D538G PIK3CA H1047R | 5.04 | Fulvestrant + alpelisib | PR* | 47.71 |
| 873 | 41 | Y | 0 | - | 3 | FM1 | AKT E17K CDK6 amp EPHB4 amp HGF amp TP53 K132R | 0 | Anti-PD-L1 + AKT inhibitor + PARPi | SD | 19.43 |
| 854 | 61 | N | 60 | - | 3 | FM1 | BRAF D594N CDH1 N174fs*41 KEAP1 M503I MCL1 amplification NF1 S1567* NF1 splice site 3314+1G>C PIK3C2B amplification PIK3CA E542K PIK3CA I1058L PTEN PTEN-HIVEP3 truncation RBM10 Q79* | 16.39 | Pembrolizumab | PR | **44.86*** |
| 0907 | 54 | Y | 0 | + | 6 | FM1 | ERBB2 amplification LYN amplification MYC amplification NOTCH2 NOTCH2 truncation PIK3CA H1047R RAD21 amplification RPTOR amplification TP53 P278R | 6.30 | STAT3 inhibitor+ trastuzumab | PD | 6.0 |
|  |  |  |  |  |  |  |  |  | Tucatinib + capecitabine + trastuzumab | PR | 21.0 |
|  |  |  |  |  |  |  |  |  | Carboplatin + gemcitabine + trastuzumab | PD | 6.43 |
| 966 | 63 | N | 80 | - | 6 | FM1 | GENE ALTERATION AKT1 amplification AKT1 E17K MSH2 Q337* TP53 V157fs*30 CDK12 Q937fs*18 MST1R I1113M ERRFI1 R244Q | 18.91 | Pembrolizumab | CR | **59.71*** |
| 968 | 37 | Y | 90 | - | 4 | FM1 | ARFRP1 amplification AKT2 amplification RAD21 amplification CDK4 amplification GNAS amplification PIK3CA E545K | 3.78 | Anti-PD-L1 + AKT inhibitor + PARPi | SD | 32 |
| 1058 | 58 | N | 90 | - | 3 | FM1 | FGF4 amplification MYC amplification CCND1 amplification FGF3 amplification FGF19 amplification MCL1 amplification PIK3CA H1047L | 3.78 | Fulvestrant + alpelisib | SD | **64.71 *** |
| 1150 | 63 | Y | 10 | - | 2 | FM1 | MDM2 amplification CDH1 Q255* RB1 L331fs*9 ARID1A K2146fs*55 KEL M1T AKT1 E17K PIK3CA H1047R PIK3CA E545K ATRX K589fs*32 | 17.65 | Anti-PD-L1 + AKT inhibitor + PARPi | PR | **36.57*** |
| 893 | 65 | Y | 90 | - | 9 | FM1 | EPHA3 amplification MAP3K1 T1231fs*8 PIK3CA H1047R PIK3CA E453K ESR1 L536Q MAP3K1 N1079fs*2 | 6.30 | Anti-PD-L1 + AKT inhibitor + PARPi | PR | **34.0 *** |
| 1195 | 52 | N | 90 | - | 1 | FM1 | RAD21 amplification FGF3 amplification MYC amplification FGF4 amplification CCND1 amplification FGF19 amplification WHSC1L1 amplification FGFR1 amplification BCL2L1 amplification ZNF703 amplification PIK3CA Y1021H PIK3CA E542K | 3.78 | Fulvestrant + alpelisib | SD | 39.43 |
| 1290 | 40 | Y | 60 | - | 1 | FM1 | FGF4 amplification CDK4 amplification RAD21 amplification BRAF amplification CCND1 amplification FGF19 amplification MYC amplification NKX2-1 amplification KEL amplification MDM2 amplification NFKBIA amplification FGF3 amplification CREBBP CREBBP truncation AKT1 E17K TP53 R248W SMAD4 S485* | 1.26 | *AKT inhibitor^+^* | PD | 2.57 |
| 1224 | 67 | Y | 90 | - | 7 | FM1 | RAD21 amplification FGF3 amplification MDM2 amplification ERBB3 amplification CCND1 amplification FGF19 amplification ESR1 amplification MYC amplification FGF4 amplification CDK4 amplification MCL1 amplification PIK3CA E542K PIK3CA M1043I | 1.26 | Anti-PD-L1 + AKT inhibitor + PARPi | SD | **8.71 *** |

**Supplementary Table 1.** Matched treatment events for subjects with *PIK3/AKT/PTEN* alterations. Each row represents an individual subject. Where one subject has multiple matched treatment events, best response, and progression free survival (PFS) are reported for each treatment event. Mechanistic description of agents may be included where investigational therapy is administered as part of a clinical trial.

ACH v2, Ampliseq Cancer Hotspot v2; CDK, cyclin dependent kinase; ER, oestrogen receptor; HER2, human epidermal growth factor receptor 2; FM1, FoundationOne; NR, not reported; PARPi, poly-ADP ribose polymerase inhibitor; PD-L1, programmed death-ligand 1; STAT3, signal transducer and activator of transcription 3; TMB (mt/Mb), tumour mutational burden (mutations/megabase); tx, treatment. *Censored at last review, no documented progression event. ^#^Matched therapy after enrolment and sequencing. ^+^Patient progressed during lead in phase of AKT inhibitor as part of clinical trial and did not receive planned combination anti-PD-L1 and PARPi.

| **Study ID** | **Age** | **De-novo MBC** | **ER %** | **HER2 status** | **No. of prior lines** | **Sequencing platform** | **Sequencing results** | **TMB** | **ICI therapy** | **Best Response** | **PFS (weeks)** |
| --- | --- | --- | --- | --- | --- | --- | --- | --- | --- | --- | --- |
| **Matched TMB** | | | | | | | | | | | |
| 641 | 65 | N | 70 | - | 7 | FM1 | CCND1 amplification  ESR1 E380Q, Y537C  PTCH1 R607T  MEN1 I85fs*33  EPHA5 E279K  FGF19 amplification⧺  FGF3 amplification⧺  FGF4 amplification⧺  RB1 R255*  SPEN E1043*, E431*  TBX3 M322I  TET2 R550Q  TGFBR2 R537C  TP53 R248Q | 24.00 | Anti-CTLA-4 + Anti-PD-1 | SD | 24.6 |
| 827 | 67 | Y | 90 | + | 10 | FM1 | PIK3C2B amplification  MDM4 amplification  ERBB2 amplification  ESR1 amplification  CCND1 amplification  ZNF217 amplification  FGF4 amplification  FGF3 amplification  FGF19 amplification  GATA3 W329fs*23  TP53 R273P  GATA3 P409fs*99  CBFB splice site 165+1G>C | 18.91 | Pembrolizumab | PD | 6 |
| 854 | 61 | N | 60 | - | 4 | FM1 | BRAF D594N  CDH1 N174fs*41  KEAP1 M503I  MCL1 amplification  NF1 S1567*  NF1 splice site 3314+1G>C  PIK3C2B amplification  PIK3CA E542K  PIK3CA I1058L  PTEN PTEN-HIVEP3 truncation  RBM10 Q79* | 16.39 | Pembrolizumab | PR | 44.9* |
| 966 | 63 | N | 80 | - | 7 | FM1 | GENE ALTERATION  AKT1 amplification  AKT1 E17K  MSH2 Q337*  TP53 V157fs*30  CDK12 Q937fs*18  MST1R I1113M  ERRFI1 R244Q | 18.91 | Pembrolizumab | CR | 59.7* |
| 1150 | 63 | Y | 10 | - | 2 | FM1 | MDM2 amplification  CDH1 Q255*  RB1 L331fs*9  ARID1A K2146fs*55  KEL M1T  AKT1 E17K  PIK3CA H1047R  PIK3CA E545K  ATRX K589fs*32 | 17.65 | Anti-PD-L1 + AKT inhibitor + PARPi | PR | 36.6* |
| **Unmatched for TMB** | | | | | | | | | | | |
| 098 | 64 | N | 0 | - | 7 | ACH v2 | AKT1_E17K | - | Anti-PD-L1 | SD | 15.14 |
| 228 | 42 | Y | 100 | - | 10 | ACH v2 | No mutations | - | Anti-PRL-3 | PD | 6.14 |
| 277 | 63 | N | 5 | - | 5 | ACH v2 | PIK3CA_E542K, TP53_E286G | - | Pembrolizumab | PD | 7 |
| 377 | 46 | N | 0 | - | 1 | ACH v2 | PTEN_R233*, RB1_Q762*, TP53_R213*, SMAD4_R445*, SMARCB1_T72K | - | Pembrolizumab | CR | 209.4* |
| 565 | 63 | Y | 90 | - | 6 | FM1 | CCND1 amplification  VEGFA amplification  KDM5A amplification  WHSC1L1 amplification  PTEN loss  FGF3 amplification  MDM2 amplification  ESR1 amplification  CDK4 amplification  BRAF amplification  FGFR1 amplification  FGF4 amplification  FGF 19 amplification  GATA3 p409fs*99 | 5.04 | Anti-PD-L1 + AKT inhibitor + PARPi | PD | 5.6 |
| 594 | 57 | Y | 0 | - | 6 | FM1 | PIK3CA E545K  BRAF V600E  FANCD2 I1155fs*18  GATA3 R367L  NUP93 E14K  TP53 V272M | 9.00 | Anti-PD-L1 | PR | 117.7* |
| 599 | 54 | Y | 90 | - | 9 | FM1 | PTEN loss  FGFR1 amplification⧺  MDM2 amplification  ABL1 K609del  BCL2L1 amplification⧺  FRS2 amplification  RB1 S391*  ZNF217 amplification | 9.00 | Anti-PD-L1 + AKT inhibitor + PARPi | PD | 9 |
| 733 | 67 | Y | 0 | - | 3 | FM1 | CTNNB1 Y331*  PIK3R1 E451_Y452del  TP53 R175H | 6.30 | Anti-IL-1β + Anti-PD-1 | PD | 3.4 |
| 740 | 61 | N | 0 | - | 3 | FM1 | MYC amplification  FGF12 amplification  BCL2L1 amplification  NOTCH3 amplification  RAD21 amplification  LYN amplification  CBFB E84fs*32  PIK3CA E545K  CASP8 P363fs*37  TP53 splice site 673-1G>C | 1.26 | Anti-PD-L1 + AKT inhibitor + PARPi | SD | 26 |
| 873 | 41 | Y | 0 | - | 6 | FM1 | AKT E17K  CDK6 amp  EPHB4 amp  HGF amp  TP53 K132R | 0 | Anti-PD-L1 + AKT inhibitor + PARPi | SD | 19.4 |
| 911 | 61 | N | 0 | - | 1 | FM1 | FGFR1 amplification  MAP3K1 S782*  PIK3CA Q546R  PTCH1 splice site 1602+2T>C  PTEN T319fs*1  PTEN V290fs*8  RB1 E691*  SPEN E1871*  WHSC1L1 amplification  e | 3.78 | Anti-IL-1β + Anti-PD-1+ Anti-LAG-3 | SD | 81* |
| 968 | 37 | Y | 90 | - | 4 | FM1 | ARFRP1 amplification  AKT2 amplification  RAD21 amplification  CDK4 amplification  GNAS amplification  PIK3CA E545K | 3.78 | Anti-PD-L1 + AKT inhibitor + PARPi | SD | 32 |
| 893 | 65 | Y | 90 | - | 9 | FM1 | EPHA3 amplification  MAP3K1 T1231fs*8  PIK3CA H1047R  PIK3CA E453K  ESR1 L536Q  MAP3K1 N1079fs*2 | 6.30 | Anti-PD-L1 + AKT inhibitor + PARPi | PR | 34* |
| 1224 | 67 | Y | 90 | - | 7 | FM1 | RAD21 amplification  FGF3 amplification  MDM2 amplification  ERBB3 amplification  CCND1 amplification  FGF19 amplification  ESR1 amplification  MYC amplification  FGF4 amplification  CDK4 amplification  MCL1 amplification  PIK3CA E542K  PIK3CA M1043I | 1.26 | Anti-PD-L1 + AKT inhibitor + PARPi | PD | 8.71* |

**Supplementary Table 2**. Immune Checkpoint Inhibitor (ICI) treatment events in metastatic breast cancer (MBC) patients. Mechanistic description of agents may be included where investigational therapy is administered as part of a clinical trial. ACH v2, Ampliseq Cancer Hotspot v2; CTLA-4, cytotoxic T-lymphocyte antigen 4; ER oestrogen receptor; FM1, FoundationOne; IL-1β, interleukin 1 beta; LAG-3, lymphocyte-activation gene 3; PARPi, poly-ADP ribose polymerase inhibitor; PD-1, programmed death protein 1; PD-L1, programmed death-ligand 1; TMB (mt/Mb), tumour mutational burden (mutations/megabase); PFS, progression free survival; PRL-3, phosphatase of regenerating liver 3. *Censored at last review, no documented progression event. + matched status classified as unmatched in the event of unknown TMB (CTRAD platform did not report TMB).

| Characteristic | Matched (n=36) | Unmatched (n=69) |
| --- | --- | --- |
| Mean age (range) | 55 (36-69) | 55(29-75) |
| De-novo MBC (%) | 16 (44) | 25 (36) |
| Median number of prior treatment lines | 4 | 3 |
| Prior chemotherapy (%) | 29 (81) | 55 (80) |
| ER/PR positive, HER2 negative (%) | 25 (69) | 40 (58) |
| HER2 positive (%) | 8 (22) | 9 (13) |
| TNBC (%) | 3 (8) | 20 (29) |

**Supplementary Table 3.** Comparison of characteristics for patients (n=105) who received matched therapy at any point post sequencing (matched) versus those that did not (unmatched).

Survival analysis – first line post sequencing

A non-pooled per patient analysis (n=105) based on first line treatment post sequencing was performed. Survival differences between patients receiving matched versus unmatched treatment were non-significant but appeared to favour matched therapy with mPFS 24.4 versus 14.6 weeks, HR= 0.78 (95% CI:0.49, 1.23, p=0.29) and mOS 30.1 versus 12.1 months, HR=0.52 (95% CI: 0.27, 1.01, p=0.053).
